# Collargolum in the Treatment of Infectious Diseases

**Published:** 1903-04

**Authors:** 


### ABSTRACT.

### Collargolum in the Treatment of Infectious Diseases.

Professor Netter, of Paris (*Bulletins et Mémoires de la Société Médicale des Hopitaux de Paris*, No. 37, December 18, 1902), details at length eleven cases treated with endovenous injections of collargolum and inunctions of unguentum Credé, with excellent results, all of them recovering rapidly. They consisted of one acute pericarditis, one pneumonia with purulent effusion; one cerebrospinal meningitis; a grave complicated scarlatina; two severe diphtherias; an acute ulcerative tuberculosis; three cases of typhoid; and one pyemia.

The favorable action of collargolum is first evidenced by the fall in temperature, which occurs either suddenly or gradually. This is particularly prompt when the medication is administered early. At the same time occurs a remarkable improvement of the general condition of the patient, the quick return of strength and appetite recalling the phenomena so often noticed after the injection of antitoxin.

The local changes are sometimes equally rapid. By the next day phlegmons and gland irritations have lost their inflammatory character, and swellings are often so diminished that only traces of them remain. Ganglionic enlargements promptly resolve; pleuritic suppurations are absorbed; pericarditis and pneumonia are favorably influenced; and even valvular lesions disappear.

Based on his observations, Netter recommends collargolum in pyemia, septicemia, in all the various forms of puerperal infection, in infectious endocarditis, cerebrospinal meningitis, scarlatina, mixed diphtheria, grave typhoid fever, in certain tuberculoses of the pneumonic form, in pneumonia, and in rheumatism with a tendency to visceral complications. In most cases inunctions of the Credé ointment suffice for the introduction of the medicament into the system. But when an immediate and vigorous action is urgently needed, recourse to intravenous injections should be taken.

